# White-Tailed Deer Vigilance: The Influence of Social and Environmental Factors

**DOI:** 10.1371/journal.pone.0090652

**Published:** 2014-03-05

**Authors:** Marcus A Lashley, M. Colter Chitwood, Michael T. Biggerstaff, Daniel L. Morina, Christopher E. Moorman, Christopher S. DePerno

**Affiliations:** Department of Forestry and Environmental Resources, North Carolina State University, Raleigh, North Carolina, United States of America; Federal University of Parana (UFPR) ) – Campus Palotina, Brazil

## Abstract

Vigilance behavior may directly affect fitness of prey animals, and understanding factors influencing vigilance may provide important insight into predator-prey interactions. We used 40,540 pictures taken withcamera traps in August 2011 and 2012to evaluate factors influencing individual vigilance behavior of white-tailed deer (*Odocoileus virginianus*) while foraging at baited sites. We used binary logistic regression to determine if individual vigilance was affected by age, sex, and group size. Additionally, we evaluated whether the time of the day,moon phase,and presence of other non-predatorwildlife species impacted individual vigilance. Juveniles were 11% less vigilant at baited sites than adults. Females were 46% more vigilant when fawns were present. Males and females spent more time feeding as group size increased, but with each addition of 1 individual to a group, males increased feeding time by nearly double that of females. Individual vigilance fluctuated with time of day andwith moon phase but generally was least during diurnal and moonlit nocturnal hours, indicating deer have the ability to adjust vigilance behavior to changing predation risk associated with varyinglight intensity.White-tailed deer increased individual vigilance when other non-predator wildlife were present. Our data indicate that differential effects of environmental and social constraints on vigilance behavior between sexes may encourage sexual segregation in white-tailed deer.

## Introduction

Non-consumptivepredation effects can impact interactions of prey specieswith their environment and may negatively affect fitness [1].High predation risk may reduce fitnessby simplifying an animal’s decision making rules,which potentially hinders optimal use of resources,particularly when foraging areas are separated from escape cover [2]. Therefore,predation risk may have an impact on prey behaviorsand subsequently affect fitness of the population [3]. Behaviors that potentially reduce fitness may include reduced feeding durations [4], [5], decreases in dielactivity [6], changes in group size [7], [8], changes in habitat use [9]–[12], and increases in vigilance while foraging [13].

During feeding,vigilance behavior comes at a cost if intake rates are decreased [14]–[16]. However, ungulates generally accept the cost of vigilance during foraging because the cost of decreasing intake is a lower proximal threat to the individualfitness than increased predation risk [7]. Therefore, individual vigilance should be positively correlated to perceived predation risk [17]. Furthermore, individuals may increasegroup sizes to decrease individual vigilance during foraging without increasing predation risk [8].

The predation risk hypothesis predicts that larger male ungulates are less vulnerable to predation than females and young, and males select areas with higher quality resources and greater predation risk, whereas females select lower quality patches where predation risk is less [18], [19]. Similarly, the predation risk allocation hypothesis predicts animals will respond to pulses in predation risk by allocating more time to anti-predator behaviors, and animals will respond quickly to fluctuations in risk [20], [21]. Both hypotheses may explain sexual segregation of sexually dimorphic ungulates [18], [19]. Because white-tailed deer (*Odocoileus virginianus*) are sexually dimorphic, males and females should perceive different predation risks and diverge in anti-predator behaviors and ultimately segregate [22]. However, anti-predator behavior may be influenced by factors other than sex,including reproductive status, social rank, and group size [23]–[27].Additionally, changing light intensity [28] and interspecific changes in group size (i.e., shared vigilance; [29])may affect predation risk perception and consequently alter anti-predator behavior.

We investigated potential factors that influence individual vigilance of foraging white-tailed deer anddetermined if sex, age class, andgroup size influenced vigilance behavior.We expanded on Lark and Slade [23] by using camera traps, which allow the evaluation of vigilance relative to time of day,moon phase, and the presence of other non-predator wildlife species and provide the opportunity to collect large volumes of data while minimizing the potential bias of human presence. Assuming predator density was fairly homogeneous across the study site, we hypothesized males would be less vigilant than females because of larger body size, juveniles would be less vigilant than adults because of inexperience, both sexes would decrease individual vigilance with increasing group size, and vigilance would be greatest during the brightest times of the day and night (i.e., full moon), when their predators presumably had the best eyesight conditions. Furthermore, we hypothesized the presence of other non-predator wildlife species would decrease vigilance behavior by increasing the interspecific group size.

### Ethics Statement

This research was performed in accordance with the United States Department of Defense and Fort Bragg Military Installation research permit. No animals were handled in this study.The funders designed the sampling scheme and camera trap positions based on proper techniques for white-tailed deer population surveys as indicated by previous literature; however, the funders had no role in data collection and analysis, decision to publish, or preparation of the manuscript.

## Materials and Methods

### Site Description

We conducted our study on Fort Bragg Military Installation (Fort Bragg), located (35°7’ N, 79° 9’ W) within the Sandhills physiographic region in the lower coastal plain of North Carolina, USA. Forests were managed with growing-season prescribed fire on a 3-yr fire-return interval. Upland forests weredominated by longleaf pine (*Pinus palustris*) with wiregrass (*Aristidabeyrichiana*)understories [30]. Potential predators of deer included coyotes (*Canis latrans*), bobcats (*Lynx rufus*), and humans. Deer werehunted in accordance with state game regulations from the first Saturday in September to 1 January each year.The large area and fairly homogeneous land management across Fort Bragg allowed us to uniquely isolate the effects of social and environmental variability on vigilance behavior.

### Data Collection

In August of 2011 and 2012, we established 100 baited sites(i.e., 50 sites established August 1and 50 sites established August 8).We chose to conduct our study in August because the effects of hunting on vigilance were minimized and we could monitor pre-harvest population density and fawn recruitment as described in Jacobson et al. [31]. We set sites in a grid design across Fort Bragg so there was 1 camera per 500ha (i.e., 50,000ha ÷ 100 sites), which is much larger than the reported summer home range (40–90ha) of white-tailed deer [32]. We pre-baited for 14 days and then activated cameras to take pictures for 14 days and as frequently as every 3 minutes [31]. Cameras were triggered by motion and heat and were equipped with infrared flash to reducestartling deer during nocturnal hours. After the 14 days of camera trapping, we collected all pictures and tallied the number,sex, and age of deer, their vigilance level, the time and date of the picture (rounded to the nearest hour), presence of other wildlife species, and the moon phase (New, First quarter, Full, Third quarter, as described in Rockhill et al. [33]).We considered a deer to be in a feeding posture if its head was below its stomach line (non-vigilant)and classified it as non-feeding posture when its head was above the stomach line. We determined sex based on the presence or absence of antlers; if the head was not visible the picture was discarded. We classified deer into 1 of 2 age classes, juvenile (<1yr) and adult (>1yr), based on the presence or absence of spotted pelage, respectively. Each picture had a time and date stamp, sowe acquired daily fraction of the moon illuminated (from the Naval Oceanography Portal; http://www.usno.navy.mil/) and moon phase.

### Data analysis

We used a binary logistic regressionmodel in SPSSto analyze factors affecting the time spent in the non-vigilant feeding posture. We setposture as the binary dependent variable with feeding posture or non-feeding posture (feeding posture  = 1 and non-feeding posture = 0) being the possible outcomes. Age (Adult = 1 and Juvenile = 0), sex (Male = 1 and Female = 0), group size, time, presence or absence of other wildlife species (Absent = 1 and Presence = 0), and moon phase were set as independent variables in the model; we included all interactions between sex and other independent variables and the moon phase × time of day interaction. We set alpha to 0.05. Also, we reported the time spent non-vigilant (i.e., pictures in feeding posture ÷ total number of pictures). Because some of the time spent in non-feeding postures could be spent handling forages (or otherwise non-vigilant; [34]), our assessment of time spent foraging is a conservative estimate of actual foraging behavior.

## Results

**Figure 1:**
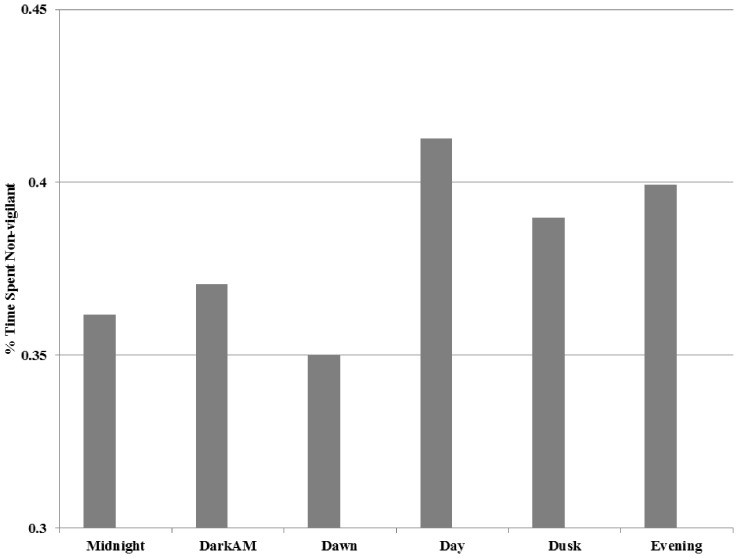
Influence of time of dayon time spent non-vigilantby white-tailed deerwhile at baited sites at Fort Bragg Military Installation, North Carolina, USA, August 2011 and 2012. Time spent feeding was greater in the post-meridiem(P = 0.03).

**Figure 2:**
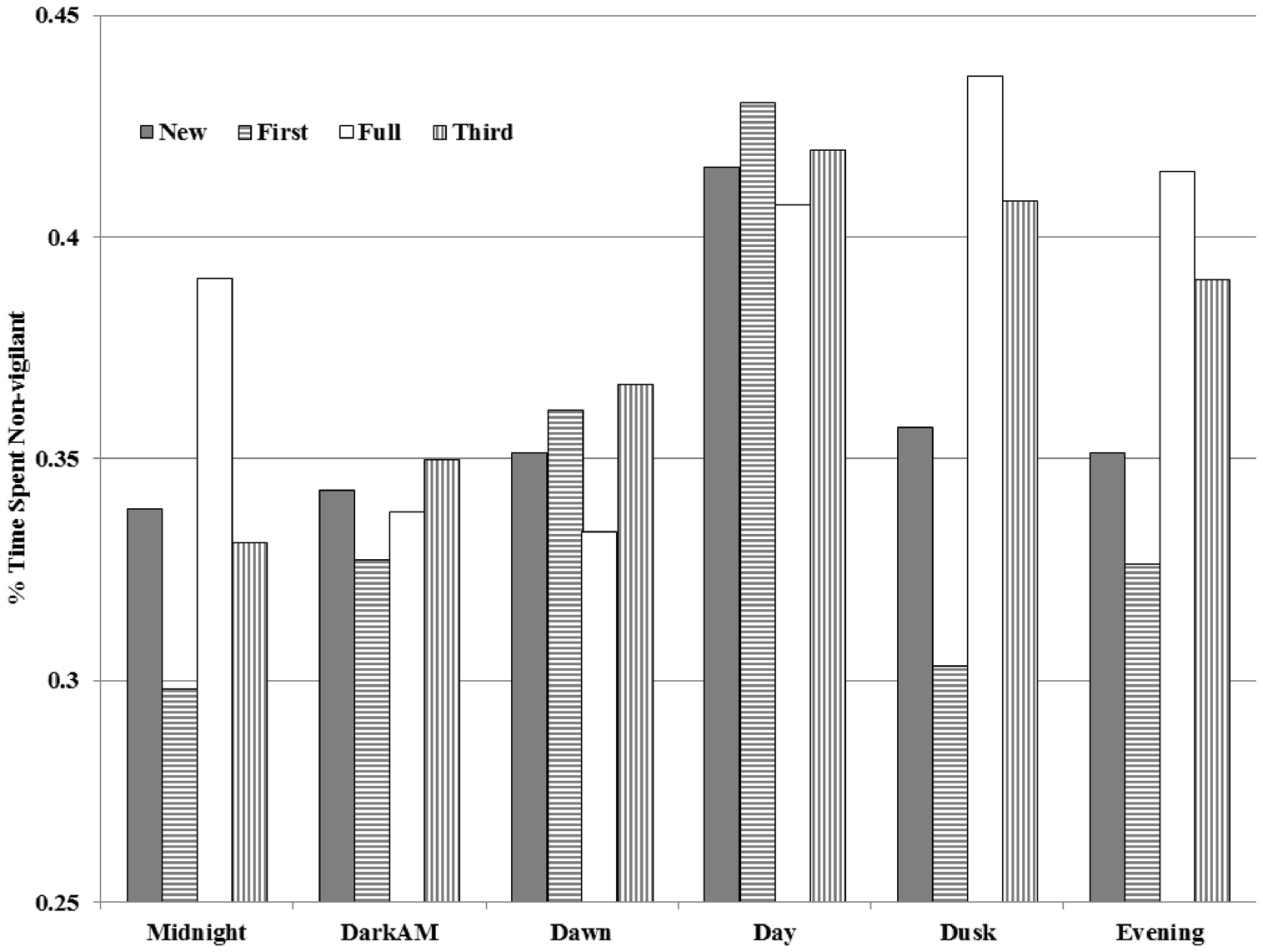
Influence of moon phase and time of day on time spent non-vigilant by white-tailed deer while at baited sites at Fort Bragg Military Installation, North Carolina, USA, August 2011 and 2012. Time spent feeding was greater during moonlit nocturnal hours(P<0.001).

**Figure 3:**
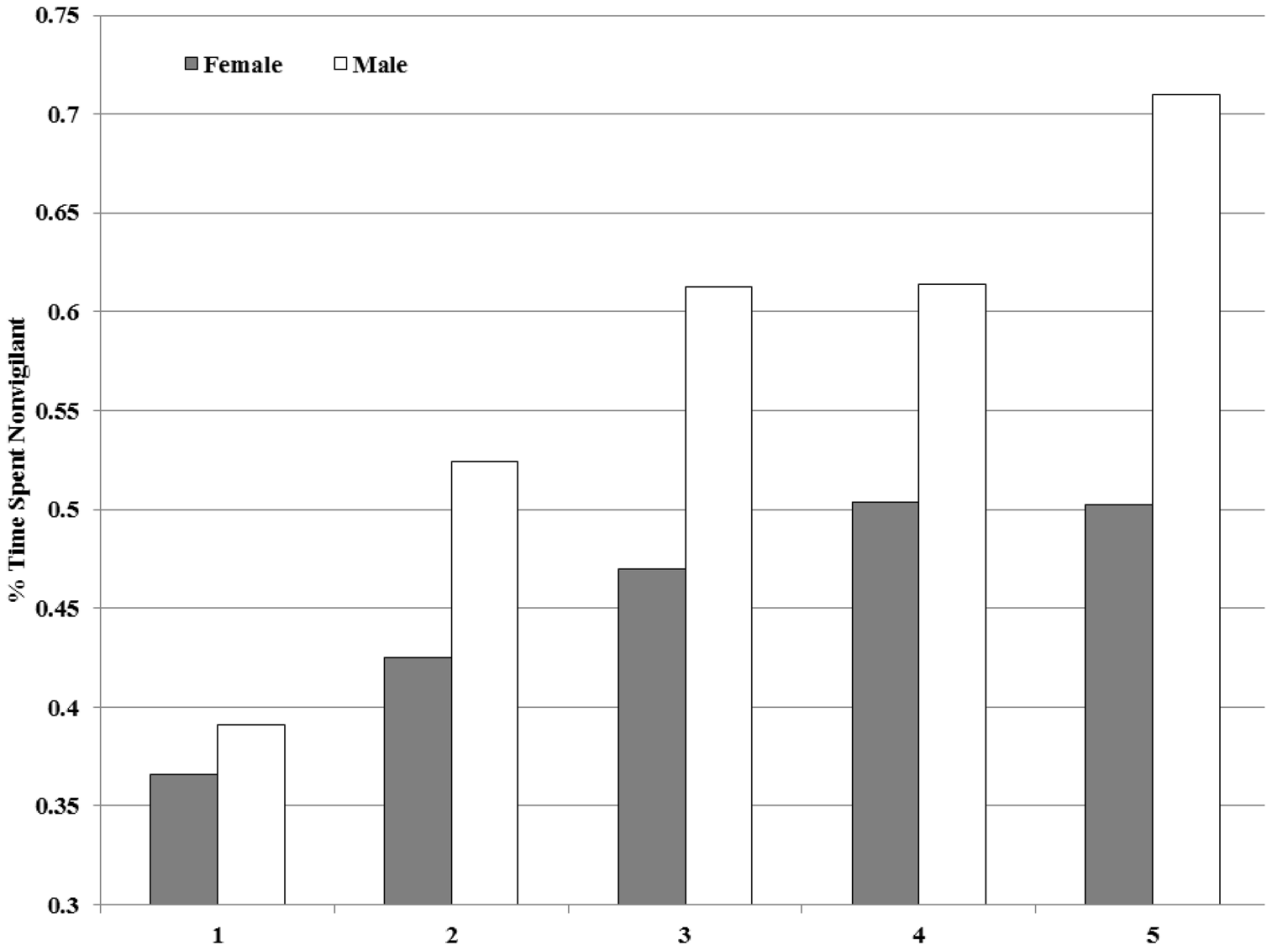
The influence of group size on time spent non-vigilantby female and male white-tailed deer while at baited sites at Fort Bragg Military Installation, North Carolina, USA, August 2011 and 2012. Time spent feeding increases with each addition of group size but males increased at twice the rate per addition to group size(P<0.001).

We collected 40,540 photographs of deer. We discarded 234 pictures because of inability to determine sex, age class, or posture. Pictures were relatively homogeneously distributed among cameras with only one camera site failing to receive any deer use. Less than 1000 pictures wereconfined to any single camera site per year,whichminimized the weight of unique behavior at any camera site throughout the study. We recorded 24,934 pictures of females, 15,372 of males, 17,567 pictures with a group size of 1, 14,285 in group sizes of 2, 6,194 in groups of 3, and the remainder in group sizes of 4 or larger (largest group = 7). Males were 20% less vigilant than females while foragingat baited sites (Table 1). Deer were less vigilant in the post-meridiem (Table 1, Figure 1) and less vigilant during brighter moon phases (Table 1, Figure 2). Both males and females spent more time feeding while at baited sites as group size increased, but with each addition of 1 individual to a group, males increased feeding time by nearly double that of females (7% per individual in females and 15% per individual in males)(Table 1, Figure 3). Males continued to increase feeding timeup to group sizes of 5, but females did not increase feeding time in groups larger than 4 (Table 1, Figure 3).Females were 46% more vigilant when fawns were present (P<0.001), and juveniles were less vigilant at baited sites than adults, averaging11% less vigilant behavior(Table 1).Also, males and females were 10% more vigilant when other wildlife species were present (Table 1).

**Table 1:** Parameters of the binary logistic regression model to predict feeding posture of white-tailed deer at baited sites at Fort Bragg Military Installation, North Carolina, USA, August 2011 and 2012.

	β	Standard Error	Exp(β)	Wald Statistic	DF	P-value^a^
Intraspecific group size	0.321	0.014	1.378	495.735	1	<0.01
Sex	0.433	0.024	1.542	338.03	1	<0.01
Lunar phase	0.037	0.008	1.038	19.047	1	<0.01
Presence of other wildlife	0.337	0.024	1.401	196.819	1	<0.01
Age	–0.496	0.04	0.609	157.304	1	<0.01
Time of day	–0.265	0.011	0.768	69.465	23	0.03
Constant	–0.968	0.124	0.38	60.923	1	<0.01

a Alpha was set to 0.05.

## Discussion

Our results support the premise that sexual segregation of white-tailed deer may be based on differences in risk perception between sexes [18], [21], [22], [35]. Males were less vigilant at baited sites, likely because they perceive lowerpredation risk by natural predators due to their larger body size compared to females [18].Variation in risk perception potentiallychanges feeding durations [4], [5], daily movement [6], and group sizes [8],and these changes may contribute to sexual segregation without requiring resource partitioning or competitive exclusion of one sex over the other [36].

Deer were less vigilantduring brighter periods, likely because they were better able to see predators. However, this is inconsistent with other prey species that secondarily use eyesight to detect predators [28]. Though deer eyesight is fairly poor in comparison to other senses (e.g., smell and hearing; [37]), eyesight is important in determining the intent of an approaching predator [38]. Whereas, olfactory cues may be important for establishing overall predation risk [39], we assumed scent deposition from predators was not correlated with light intensity. Therefore, diurnal and moonlit nocturnal hours likely allow a greater visual capability with additional information about predation risk, which allowsdeer to be less vigilant while foraging.

Time spent feeding increased as group size increased, likely because individuals were able to spend less time scanning for predators and more time foraging without increasing predation risk per individual [40].The influence of group size on individual vigilance has been well-documented [13], [40], [41] and grouping behavior clearly affects fitness [42]. However, the magnitude of change was different between males and females,contrasting other studies that reported either little effect of group size on individual vigilance of males or less difference in magnitude between sexes as group size increases [43]–[46].In sexually dimorphic cervids, sexual segregation is most pronounced during non-mating seasons when the sexes are in large groups [47]. Therefore, the difference in vigilance behavior between the sexes we observed as a result of group size coupled with other social dynamic constraints could be a causal mechanism for the ubiquitous sexual segregation of dimorphic ruminants outside the mating season [47]–[48].

Greatervigilance behavior by females may confound the nutritional demands of lactation (May-August at Fort Bragg) by requiring greater time spent foraging to support the already heightened intake requirements. Because lactation is the most nutritionally demanding physiological condition in deer [49] and requires an increased forage intake rate from that of other physiological conditions [50], vigilance behavior may come at a greater cost to females during lactation. Toïgo [51] reported lactating female French Alpine ibex (*Capra ibex ibex*) had greater vigilance than their non-lactating conspecifics and offset the additional time of vigilance behavior by decreasing the time resting between foraging bouts. In areas of high predation risk, increasing movement rates could lead to a paradoxical situation whereby increased movement predisposes an individual to predation it is trying to offset by moving more often between feeding bouts; in such cases, predation risk could resultin a significant reduction in fitness. Increased vigilance during lactation may requiremore time spent foragingand increased time spent searching for food [12], [20]and decreased use of high quality food patches [51]. Concomitantly,increasing the time spent foraging and searching for foods may increase risk of predator-prey interactions,whichmay require increasedvigilance [17]. Eventually, the time budget may not allow lactating females to acquire enough resources to support lactation, which could lead to neonate starvation by abandonment coupled with substantially reduced neonate survival from predation [52].

White-tailed deer do not seem to share vigilance with other wildlife species while foraging. Though interspecific increases in group size have been demonstrated to decrease individual vigilance rates in some species [53]–[55], deer may nothave the same pattern because the baited sites artificially concentrated other wildlife species that do not commonly forage togetherwith deer and because of a disparity in body size across species [29]. Though noise may not affect vigilance without some associated negative stimuli [56], the presence of non-predator species at baited sites may have increased vigilance because noise and movements of those species invoked the anti-predator response of deer.

## Conclusions

Vigilance behavior plays a major role in the acquisition of resources and predator avoidance. Our data indicate environmental and social factors influence individual vigilance. Furthermore, sexual segregation may be encouraged by differential effects of environmental and social factors between sexes. Further investigation of the influence of vigilance behavior on sexual segregation is warranted.
